# Echoed induction of nucleotide variants and chromosomal structural variants in cancer cells

**DOI:** 10.1038/s41598-022-25479-6

**Published:** 2022-12-05

**Authors:** Yusuke Matsuno, Rika Kusumoto-Matsuo, Yuya Manaka, Haruka Asai, Ken-ichi Yoshioka

**Affiliations:** Laboratory of Genome Stability Maintenance, National Cancer Center Research Institute, Tsukiji, Chuo-ku, Tokyo, 104-0045 Japan

**Keywords:** Cancer genomics, Genomic instability, Genomics, Mutation

## Abstract

Generally, the number of single-nucleotide variants (SNVs) in somatic cells increases with age, which is expected for replication errors. The number of SNVs in cancer cells, however, is often much higher than that in somatic cells, raising the question of whether cancer cells possess SNV induction pathways. The present study shows that the number of SNVs in cancer cells correlates with the number of chromosomal structural variants (SVs). While Kataegis, localized hypermutations typically arising near SV sites, revealed multiple SNVs within 1 kb, SV-associated SNVs were generally observed within 0.1–1 Mb of SV sites, irrespective of Kataegis status. SNVs enriched within 1 Mb of SV regions were associated with deficiency of DNA damage repair, including HR deficiency-associated single base substitution 3 (SBS3) and exogenous damage-associated SBS7 and SBS36 signatures. We also observed a similar correlation between SVs and SNVs in cells that had undergone clonal evolution in association with genomic instability, implying an association between genomic instability and SV-associated induction of SNVs.

## Introduction

Cancer develops through multiple rounds of clonal evolution, during which defense systems are abrogated^1–3^; this is due mainly to induction of genomic variants that include single nucleotide variants (SNVs) and chromosomal structural variants (SVs). The mechanisms responsible for induction of variants that drive cancer development remain unclear. Although it was once thought that cancer develops when variants induced in cancer-driver genes arise randomly during replication^4,5^, recent studies show that SNVs, including those in cancer-driver genes, accumulate over time, even in pathologically normal organs^6–8^. These findings suggest that these SNVs alone are not responsible for cancer development. Moreover, SNV levels are much higher in cancer cells than in cells of healthy organs^8^. This raises an important question: how are SNVs induced in cancer cells?

Recent cancer-genome studies revealed induction of a number of SBS signatures in cancer cells^9,10^. In some cases, the causes were not clear; however, in other cases, SBS signatures associated with DNA-repair deficiencies, exogenous DNA damages, and deamination were implicated^9,10^. This suggests that a type of SNV is induced in association with SVs because deamination-associated SBSs are usually induced in association with Kataegis, i.e., localized hypermutations within 1 kb of SV sites^11,12^. In addition, DNA-repair deficiencies and exogenous DNA damage are widely associated with genomic instability, which often induces SVs^13–15^.

Recent in vitro studies revealed that clonal evolution of cells with loss of ARF/p53 pathway function can be induced by genomic instability triggered by replication stress-associated DNA double-strand breaks (DSBs)^16,17^. The resulting cells show massive induction of SVs and SNVs; indeed, hotspots of SVs coincide with hotspots of SNVs^17^. Furthermore, induction of SNVs correlates strongly with induction of SVs^17^. This implies that both SVs and SNVs are induced in association with genomic instability. A potential pathway responsible for the correlation between SNVs and SVs involves high expression of low-fidelity trans-lesion synthesis (TLS) polymerases, which occurs when the genome is destabilizing^16^. The correlation between SVs and SNVs is observed within a region of 1–10 Mb^17^, a range much wider than that usually seen in Kataegis. This raises the question of whether SNVs induced in cancer cells also correlate with SVs, or simply arise due to replication errors.

The present study shows that induction of SNVs in cancer cells is associated with SVs. Peaks associated with SNV hotspots appeared as ‘echoes’ of SV peaks. Such SV-associated induction of SNVs in cancer cells was observed for both deaminase-associated and deaminase-unassociated SNVs. These results suggest that SVs are a major driver of SNV induction in cancer cells.

## Materials and methods

### Analyses of SVs and SNVs in cancer patients

The ICGC PCAWG study was searched for validated data on SVs, SNVs, insertion/deletions (indels), mutational signatures, and clinical data (sex, age at diagnosis, and survival time) in patients with breast (BRCA-EU, BRCA-UK), ovarian (OV-AU), pancreatic (PACA-AU, PACA-CA), gastric (GACA-CN), liver (LICA-FR, LINC-JP, LIRI-JP) and prostate (PRAD-CA, PRAD-UK) cancers, and in patients with pancreatic endocrine neoplasms (PAEN-IT, PAEN-AU) and chronic lymphocytic leukemia (CLLE-ES)^18^. Validated data on SVs and SNVs in mouse embryonic fibroblast cells (MEFs) were obtained from a previous study^17^.

The involvement of mutational signatures in the associations between SVs and SNV induction was analyzed using SBS signatures previously identified in each type of cancer^9^. To eliminate the effect of replication errors, mutational signatures SBS2, 13, 3, 18, 17a, 17b, 40, and 41 were analyzed after normalization with the numbers of clock-like signature (SBS1 + SBS5). Mutational signatures within 1 Mb from SV sites were evaluated with SigProfilerExtractor (https://github.com/AlexandrovLab/SigProfilerExtractor, version 1.1.4), which analyzed SNVs in these regions using default settings. The number of SVs and SNVs within 1 Mb and 100 kb windows was counted.

Circular representations of genomic alterations (SVs and SNVs) were visualized using BioCircos^19^. To analyze the effects of echoed SV/SNV-induction, the number of SVs and SNVs within each 1 Mb length of the chromosome was counted, followed by estimation of the moving averages of SVs and SNVs within 10 Mb. SV peaks were identified as local maximum inflection points within the moving averages. A “high” number of SVs and SNVs at a locus was defined as > twofold (− 20-fold) more than the genomic average and assessed by summation and superimposition of the SV and SNV data and alignments of SV peaks and SNV status. Induction of echoed SV/SNVs was analyzed at loci showing a > twofold (or fivefold) higher value than the genomic average within the ± 15 Mb flanking regions. Loci associated with Kataegis were defined as those harboring three mutations within 1 kb or more.

All SV and SNV counts analyzed in this study are listed in Supplementary Tables S1–S5.

### Estimation of random expectation for SNV induction around SV sites

To assess biased SNV induction around SV sites, SNV distributions around these sites were compared with SNV distributions based on random expectation in the whole-genome. The former was estimated based on calculation (1) below. This estimate may include genomic regions in which SVs and SNVs were not detectable by whole-genome sequencing (WGS) analyses, such as centromeres.

1 $$\mathrm{Expected\ value}=\frac{\mathrm{SNV\ number}}{\mathrm{Genome\ length}}\times \mathrm{SV\ number}\times 2\times \mathrm{Range}(1\mathrm{ k}-10\mathrm{ M})$$

## Results

### SV-associated mutagenesis (SNV induction)

**Figure 1:**
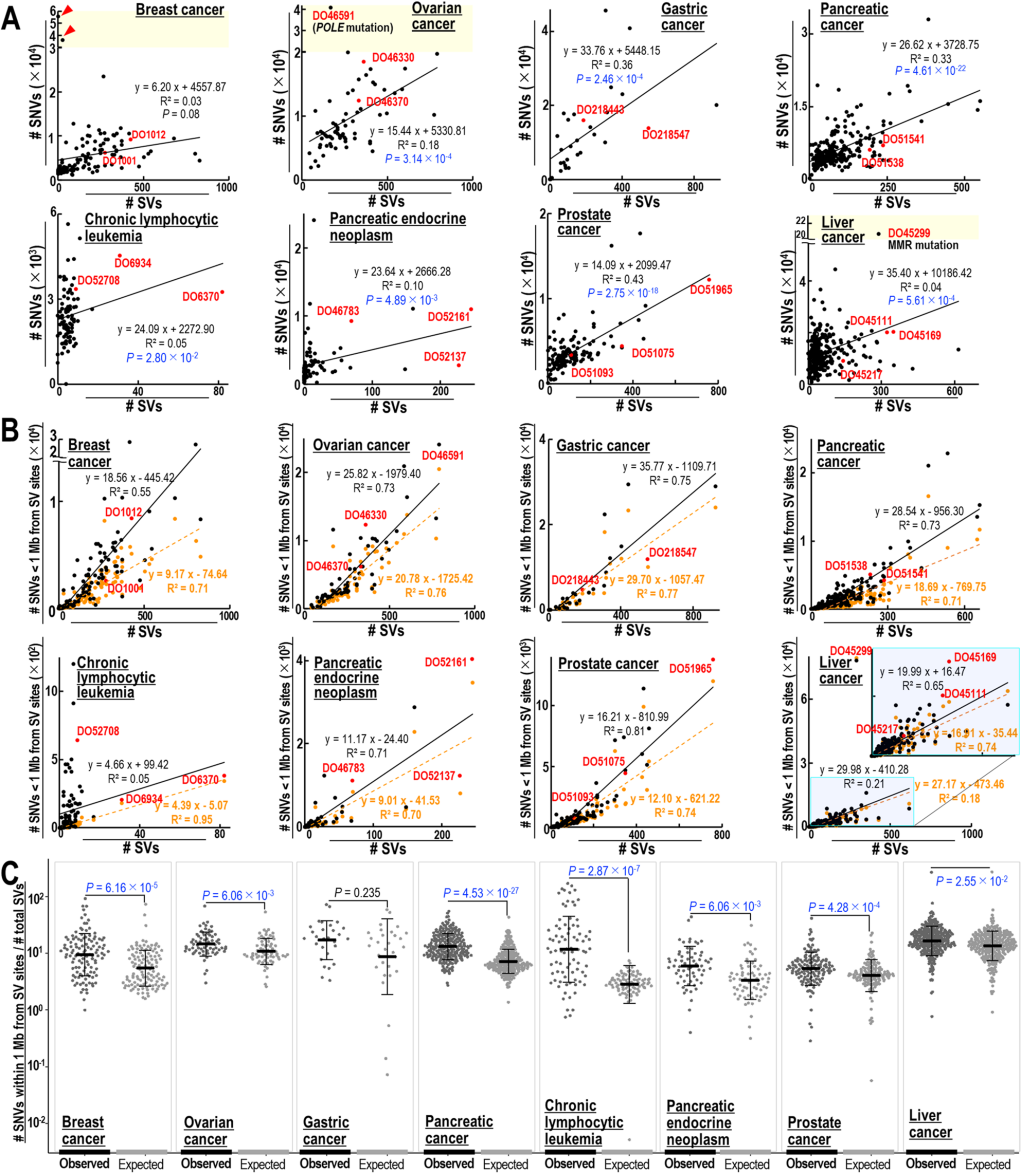
SV-associated SNV induction observed in breast, ovarian, gastric, pancreatic, prostate, and liver cancers, chronic lymphocytic leukemia, and pancreatic endocrine neoplasm. (**A**) Relationship between the numbers of SNVs and the numbers of SVs induced in individual tumors of each organ. Statistical analysis by two-tailed *t*-tests. (**B**,**C**) Comparisons of the numbers of SNVs within 1 Mb of each SV site in individual tumors of each organ with the numbers of SNVs randomly expected (**B**). Expectations were estimated based on a random distribution across detectable SNV sites within the whole genome (orange dotted line). Comparisons of relative SNV rates within 1 Mb of each SV site in individual tumors of each organ with the relative SNV rates randomly expected (**C**). Statistical analyses by two-tailed *t*-tests.

To test the possible association between SV and SNV inductions in human cancers, whole-genome sequences of eight representative human cancers were analyzed^18^. Because the patterns of SNVs in human cancers are largely affected by deaminase expression^9,10^, four types of cancer (breast, gastric, ovarian, and pancreatic cancers) frequently showing deaminase-associated SNVs (called deaminase-high cancers) and four types of cancer (chronic lymphocytic leukemia, pancreatic endocrine neoplasm, and prostate and liver cancers) rarely showing deaminase-associated SNVs (called deaminase-low cancers) were selected. SVs and SNVs were found to correlate significantly in all of these cancer types, except for breast cancer (Fig. 1A).

A previous in vitro study revealed a correlation between induction of SVs and SNVs within the 1–10 Mb range^17^. To examine SNV induction rates near SV sites in cancer cells directly, the number of SNVs within 1 Mb of an SV site was counted and compared with the random number expected (Fig. 1B,C). SNV frequencies within 1 Mb of SV sites were significantly higher than random numbers of SNVs expected in all of these cancer types, except for gastric cancer. In gastric cancers, preferential SNV induction was observed within 100 kb of SV sites (Supplementary Fig. S1A,B). These results indicate that the level of SNV induction around SV sites is generally high in both deaminase-high and -low cancers, including breast cancer. Importantly, in contrast to kataegis^11^, preferential SNV induction was observed in the genomic regions within 0.1–1 Mb of SV sites. Although the numbers of SNVs were not associated with SVs in 119 breast cancers, this may be due to the inclusion of samples from patients with multiple subtypes of breast cancer, including those showing hypermutation with few SVs. Together, these analyses revealed that SNVs are highly induced in genomic regions around SV sites.

### Age-Independent induction of SNV

**Figure 2:**
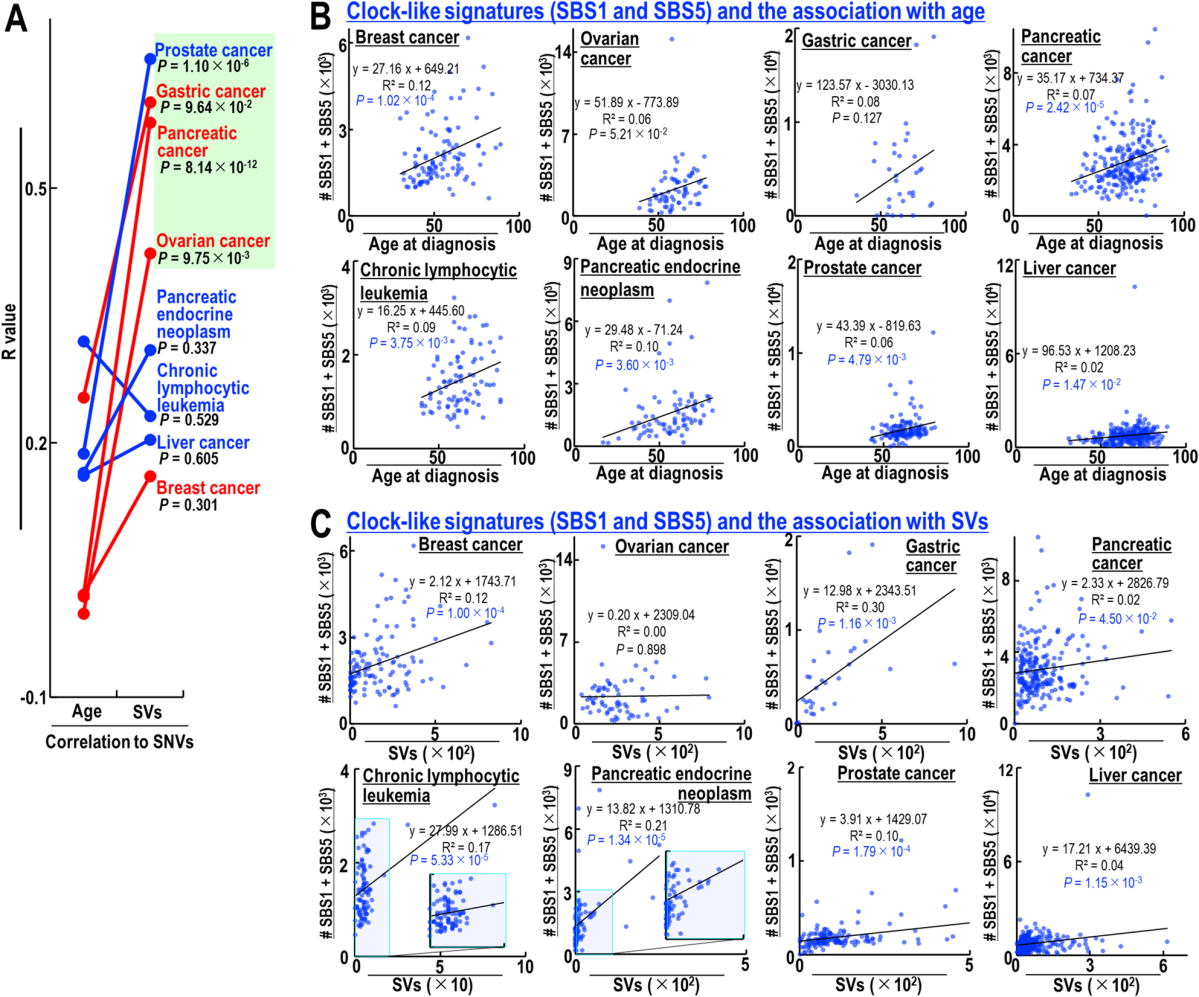
Correlated inductions of SNVs and clock-like signatures with SVs. (**A**) Comparisons of R-values (i.e., sample correlation coefficients) between SNVs and age at diagnosis and R-values between SNVs and SVs in each type of cancer. Statistical analyses by two-tailed Z-tests. (**B**,**C**) Correlations between numbers of clock-like signatures (i.e., SBS1 plus SBS5) and age at diagnosis (**B**) and numbers of SVs (**C**). Statistical analyses by two-tailed *t*-tests.

Age is a risk factor for cancer development^20,21^ and is associated with the accumulation of SNVs in healthy organs^6–8^. The association of age with the induction of SVs and SNVs was therefore tested in the eight types of cancer described above. Although age did not correlate with the rates of SNVs and SVs in deaminase-high cancers, the numbers of SNVs, but not SVs, in deaminase-low cancers increased with age, with the associations being statistically significant in patients with chronic lymphocytic leukemia and prostate and liver cancers (Supplementary Fig. S1C). Although SVs and age were associated with SNV induction (Fig. 1 and Supplementary Fig. S1C), the correlation between SVs and SNV induction was stronger than the correlation between age and SNV induction in most cancer types, and was statistically significant in patients with ovarian, pancreatic, gastric, and prostate cancers (Fig. 2A).

SNVs can be divided into several signatures according to their causes, which includes clock-like signatures SBS1 and SBS5 that increase with cell proliferation^22^. The association of clock-like signatures with age at diagnosis and SV numbers was therefore tested (Fig. 2B,C). As expected, clock-like signatures increased with age in most cancer patients, except those with gastric cancer. In addition, clock-like signatures were significantly associated with SVs in most cancer types tested, except ovarian and pancreatic cancers, indicating that even clock-like signatures are induced in a manner that correlates with SVs in both deaminase-high and -low cancers.

### Types of SNVs induced in association with SVs

Signatures other than clock-like signatures have also been observed in those cancers (Supplementary Fig. S2). The major signatures included SBS2 and SBS13, which are associated with apolipoprotein B mRNA editing enzyme catalytic polypeptide like (APOBEC), and SBS3, which is associated with homologous recombination (HR) deficiency. The association of those signatures with SVs and age at diagnosis were analyzed after normalization with clock-like signatures to reduce replication bias. Overall, those signatures rarely showed positive association with age, except for SBS18 in liver cancer, but were often positively associated with SVs, including SBS2 and SBS13 in pancreatic and liver cancers; SBS3 in ovarian, pancreatic, and prostate cancers; and SBS18 in breast, pancreatic, and prostate cancers (Supplementary Fig. S2B).

**Figure 3:**
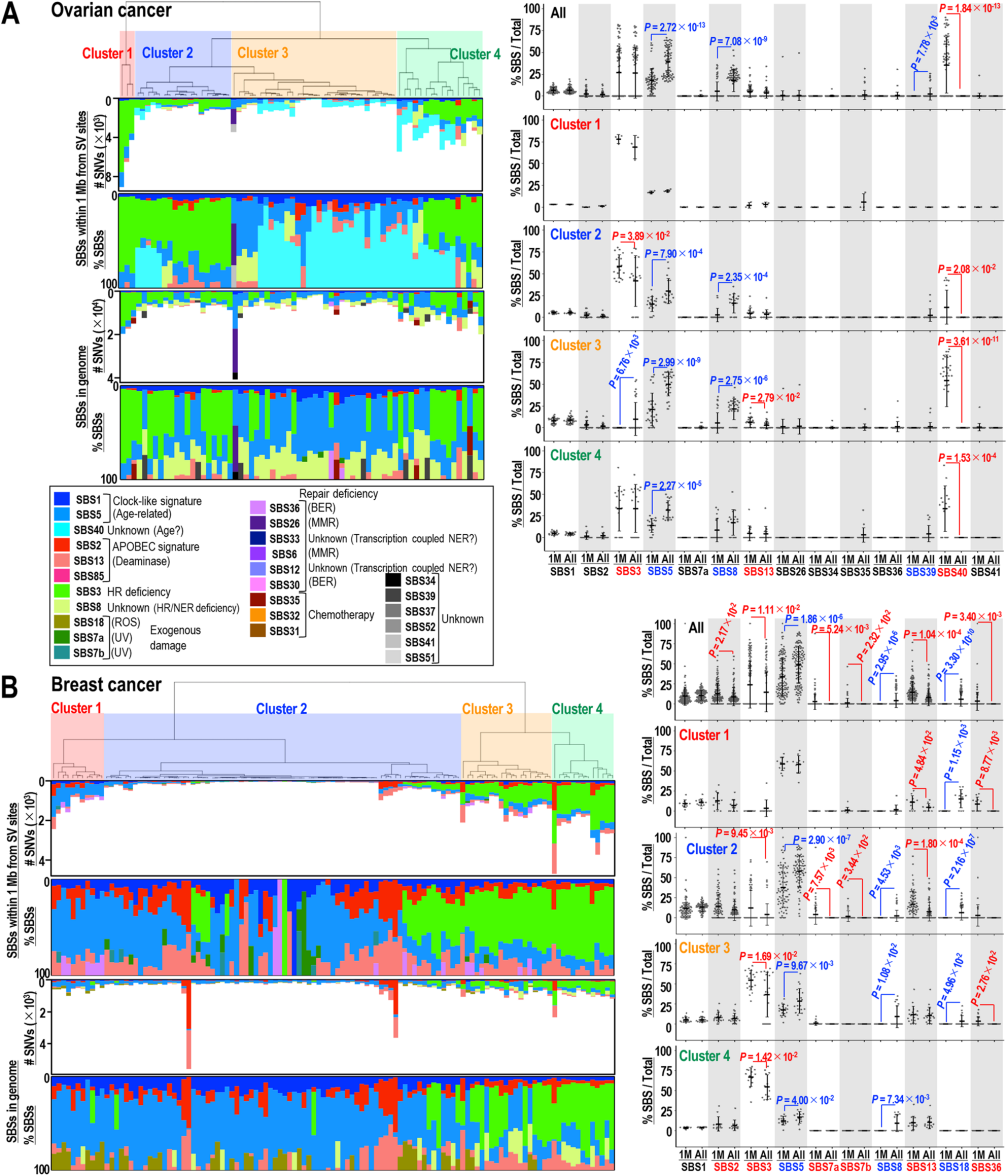
Biased inductions of APOBEC- and repair deficiency-associated signatures near SV sites in ovarian and breast cancers. (**A**,**B**) Signatures induced in ovarian (**A**) and breast (**B**) cancers within 1 Mb from SV sites (left upper panels) and in whole genomes (left bottom panels). The numbers of signatures were subsequently classified into four clusters. Percentages of SBS types induced in each tumor were plotted for all tumors and within each cluster (right panels). Statistical analyses by two-tailed Welch’s *t*-tests.

To further investigate the SNV types induced in association with SVs, we analyzed SBS signatures, as previously described^9^. Signatures accumulating within 1 Mb of SV sites were compared with those in the whole genome, especially for deaminase-high breast and ovarian cancers (Fig. 3) and deaminase-low prostate cancers and pancreatic endocrine neoplasms (Supplementary Fig. S3). Multiple signatures were found to be induced within 1 Mb of SV sites, including SBSs 2, 3, 5, 7, 13, and 40. These findings indicated the involvement of repair deficiencies, including the HR defect-associated signature SBS3; the APOBEC-associated signatures SBS2 and SBS13 (which are induced in single strand DNA usually arising during damage repair); and the exogenous damage-associated signatures SBS7a, 7b, and 36. These findings support the results showing that SNV induction is associated with SVs because SVs are caused by erroneous repair of DNA double strand breaks that can arise through exogenous damage or HR deficiency. High induction of SBS40 of unknown cause was also observed frequently. Although SBS5 is a general clock-like (age-related) signature, this signature was also induced in regions within 1 Mb of SV sites in some cancers, which is consistent with SV-associated clock-like signature induction (Fig. 2C).

### Echoed induction of SVs and SNVs

**Figure 4:**
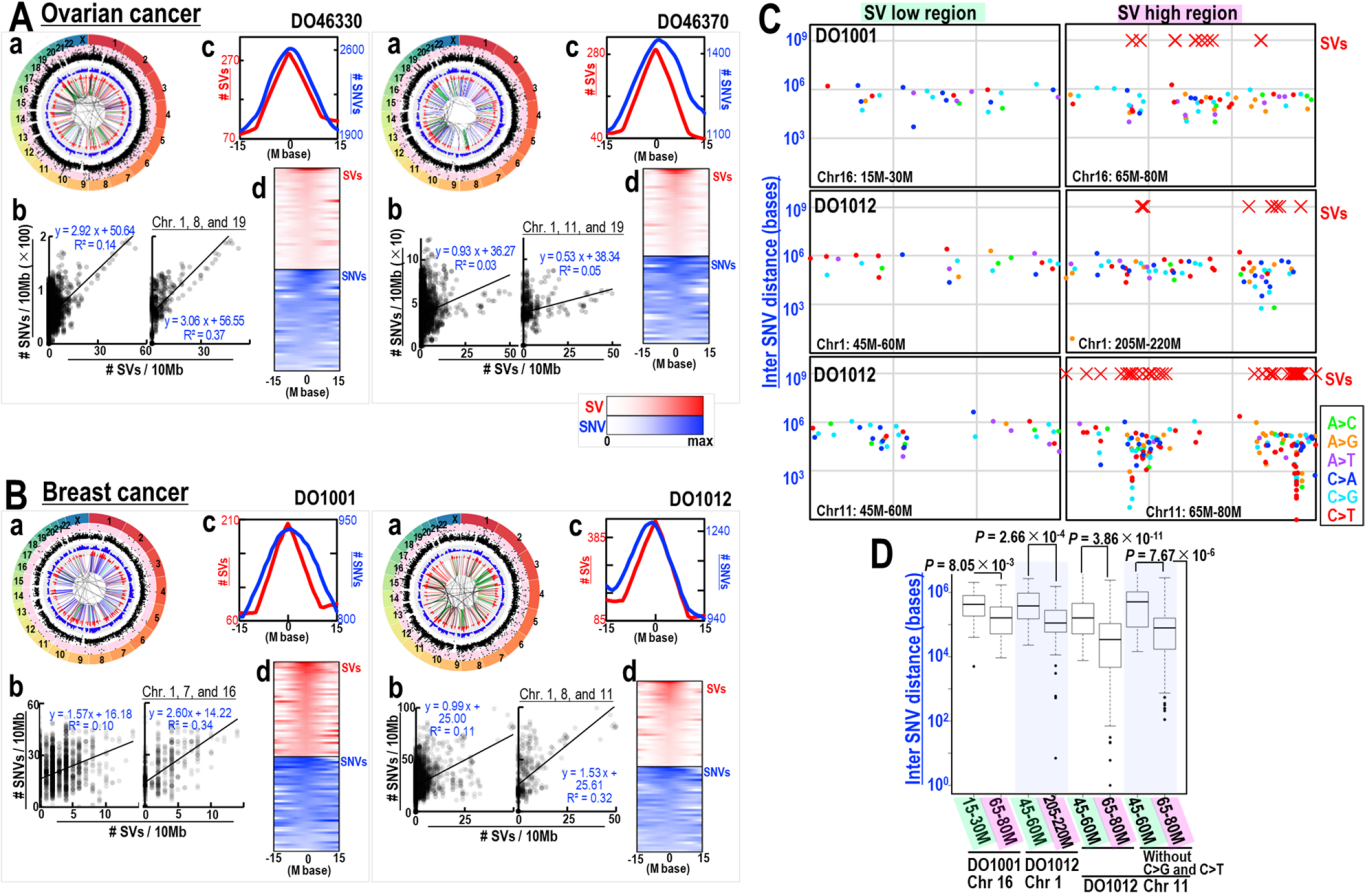
Echoed induction of SVs and SNVs in individual breast and ovarian cancers. (**A**,**B**) SVs and SNVs induced in two individual ovarian (**A**) and two individual breast (**B**) cancers. SVs and SNVs in each tumor were first analyzed by Circos plots (**a**), with chromosome ideograms shown around the outer ring. The two inner circular tracks show numbers of SVs (red) and SNVs (blue), with the corresponding moving averages. Inner lines indicate duplications (blue lines), inversions (green lines), deletions (red lines), and translocations (black lines). SNVs are also shown by rainfall plots, with black dots within pink and white circular zones indicating inter-SNV distances of < 1 kb and 1 kb–1 Mb, respectively. Correlations of SV and SNV numbers in each tumor were plotted in 10 Mb windows (**b**). Sites at which both the SV and SNV signals were more than twofold higher than the means in the genome were further analyzed by superimposing SV peaks with the associated SNV status (**c**), as well as by aligning SV peaks and SNV status (**d**). (**C**) Rainfall plots showing inter-SNV distances in breast cancers DO1001 and DO1012 along with loci at which SVs were induced; SV-low and -high regions are shown. (**D**) Box plots showing inter-SNV distances comparing these distances in SV-low and -high regions. Statistical analyses by two-tailed Welch’s *t*-tests.

The statuses of SVs and SNVs were also analyzed in individual cancer patients: samples from two patients each with eight types of human cancers were tested (Fig. 4 and Supplementary Fig. S4, see panels a and b). Samples were chosen if their results were relatively close to the correlation curves of SVs and SNVs and showed sufficient numbers of SVs for the following analyses. The statuses of SVs and SNVs in each sample was determined using Circos plots. To specifically test the associations between SV and SNV induction in each sample, these numbers were plotted in 10 Mb windows. The results showed that the inductions of SVs and SNVs were correlated in all of these samples, being more pronounced in chromosomes showing higher levels of SVs. In samples from patients with breast cancer, SNV status in SV-low and -high regions were also plotted with rainfall plots (Fig. 4C). As expected, inter-SNV distances in SV high regions were significantly smaller than those in other chromosomal regions, regardless of kataegis status (Fig. 4D). These results provided further evidence for SV-associated SNV induction in human cancer cells.

To further clarify the association between induction of SVs and SNVs, the effect of SVs on SNV signals was analyzed at sites where the frequency of SV and SNV signals was more than twofold higher than the mean frequency in the genome. As expected, SNV peaks were associated with SV peaks and these peaks resembled echoes of SV peaks (Fig. 4A and Supplementary Fig. S4, see panels c and d). Although SNV peaks in deaminase-low cancers were generally broader than those in deaminase-high cancers, the SNV peaks were still located at or near the SV peaks. Similar results were observed for the SV and SNV signal analyses conducted using a fivefold cut-off, although the number of regions showing a high number of both SVs and SNVs is smaller when the cut-off threshold is high (Supplementary Fig. S5). These results further support the notion that SNVs are generally induced in association with SVs in these cancers.

### SV/SNV status and association with prognosis

**Figure 5:**
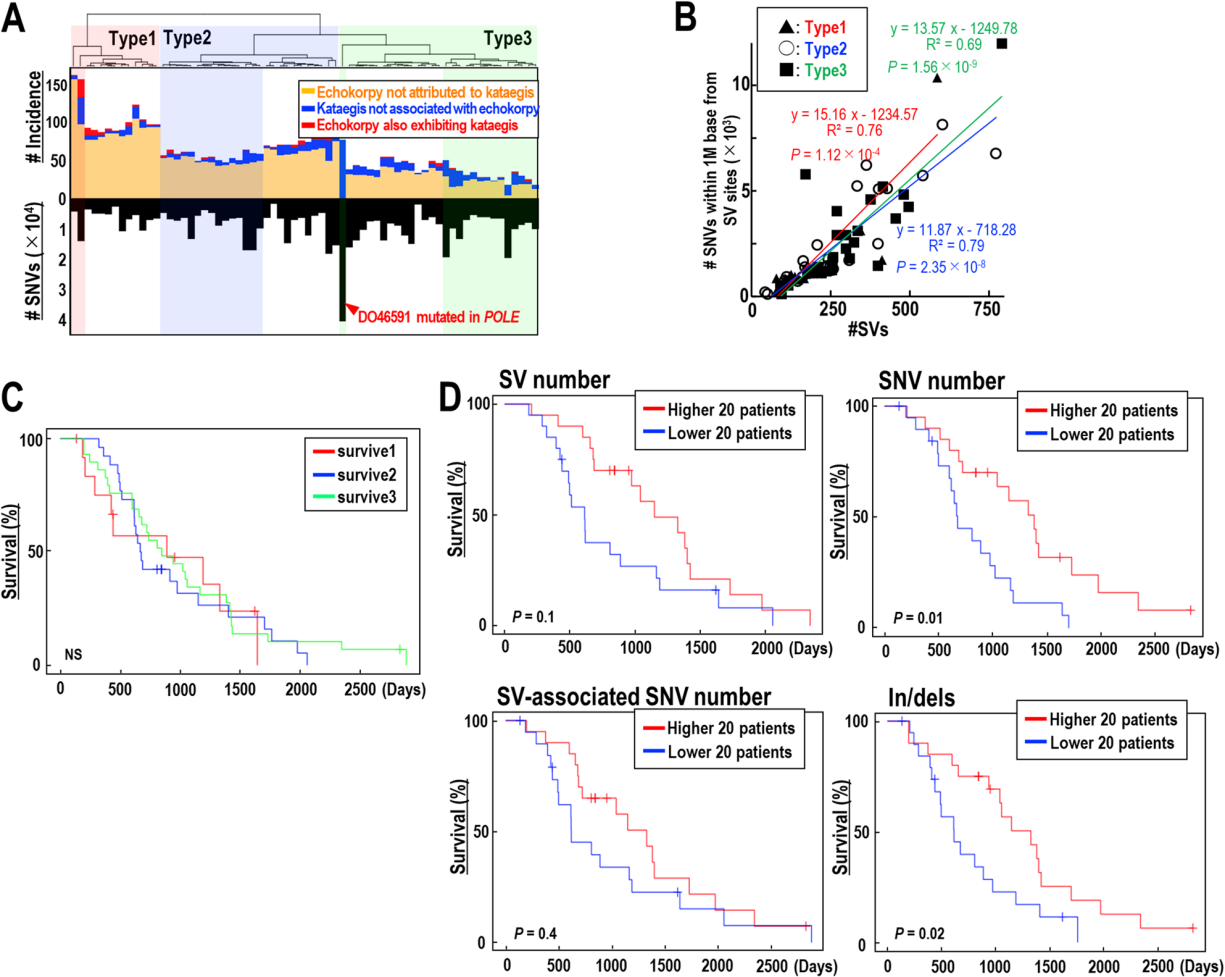
SV/SNV status of ovarian cancer and association with prognosis. (**A**) Classification of ovarian cancers according to echoed SV/SNV induction and kataegis statuses. (**B**) Relationship between SNV counts within 1 Mb of SV sites and the numbers of SVs induced in each type of individual cancers. Statistical analyses by two-tailed *t*-tests. (**C**,**D**) Kaplan–Meier analysis of survival in patients with ovarian cancer stratified by classification (**C**) and by the status of SVs, SNVs, SV-associated SNVs, and indels (**D**). Statistical analyses by log-rank tests.

Since the statuses of Kataegis and echoed SV/SNV induction varied even within ovarian cancer, we used these statuses to classify ovarian cancer types (Fig. 5A), in which SV and SNV inductions were tightly associated in all types of ovarian cancer (Fig. 5B). Although this classification did not predict prognosis (Fig. 5C), SV and SNV induction rates were associated with prognosis (Fig. 5D). Importantly, prognosis was better in ovarian cancers with higher rates of SVs, SNVs, SV-associated SNVs, and indels, with differences in SNVs and indels being statistically significant. Together with the results obtained in other cancers (Supplementary Fig. S6A–C), these findings indicate that SV/SNV induction rates are associated with clinical characteristics, such as patient prognosis. These characteristics, however, may depend on the organs in which the cancers arise. For example, SV/SNV induction rates did not correlate with prognosis in patients with pancreatic cancer (Supplementary Fig. S6A). In breast cancer, higher SV/SNV rates were likely associated with poorer prognosis, as SV and SNV rates were significantly higher in triple-negative breast cancer, which is associated with poor prognosis, than in other types of breast cancer (Supplementary Fig. S6C).

### SV-associated induction of SNVs and its association with clonal evolution

**Figure 6:**
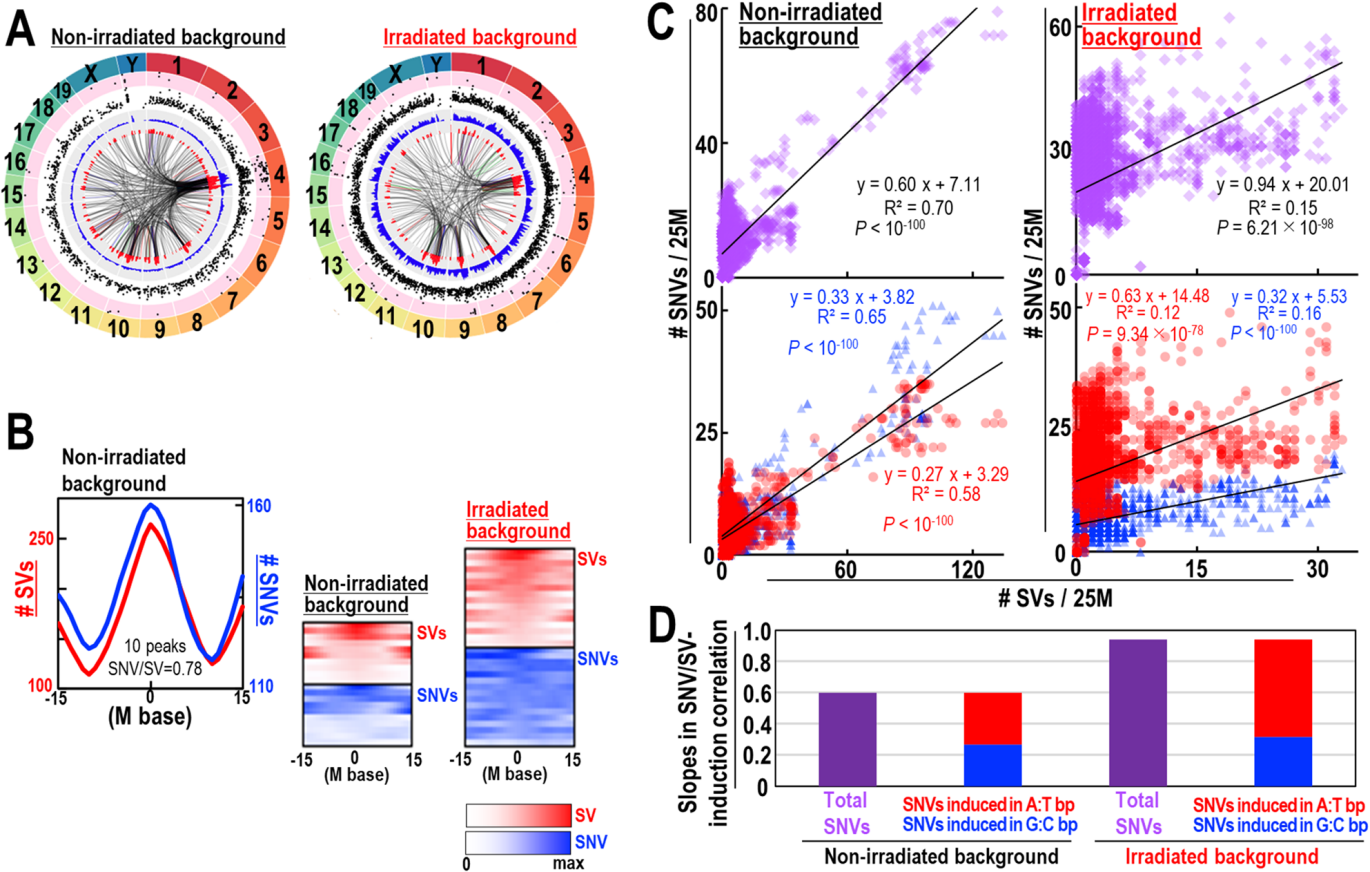
SV-associated SNV induction and association with clonal evolution. (**A**) Genome-wide Circos plots of SVs and SNVs from immortalized MEFs with and without exposure to gamma-ray irradiation (1 Gy). Chromosome ideograms are shown around the outer ring. Two inner circular tracks show numbers of SVs (red) and SNVs (blue) with corresponding moving averages. Inner lines: blue, duplications; green, inversions; red, deletions; black, translocations. SNVs are also shown as rainfall plots, with the black dots within pink and white circular zones indicating inter-SNV distances of < 1 kb and 1 kb–1 Mb, respectively. (**B**) SV peaks (red) and associated SNV statuses (blue) are superimposed and shown with peaks ± 15 Mb (top: non-irradiated) with alignment densities (bottom: 1 Gy-irradiated and non-irradiated). (**C**,**D**) Associations of SVs and SNVs in irradiated and non-irradiated immortalized MEFs. Analysis of total SNVs (**C**: top), SNVs induced in AT bp (red), and those induced in GC bp (blue) (**C**: bottom). Statistical analyses by two-tailed *t*-tests.

Recent in vitro studies using mouse embryonic fibroblasts (MEFs) revealed that clonal evolution of cells harboring mutations in the ARF/p53 pathway is caused by genomic instability; indeed, a correlation between induction of SVs and SNVs was observed in the resulting clones carrying mutations in the ARF/p53 pathway^16,17^. Here, we found that SVs/SNVs induced in cancer cells are similar to those induced by genomic instability in vitro. To examine the similarity between SVs/SNVs induced in MEFs and those induced in cancer cells, we examined SV/SNV induction peaks in MEFs (Fig. 6A,B). As expected, we observed echoed SV/SNV induction, supporting the notion that induction of SV/SNVs in MEFs is qualitatively similar to that in cancer cell genomes. Given that genomic destabilization is responsible for the resulting clonal evolution^16^, our results suggest that genomic destabilization could increase cancer risk through induction of SV-associated SNVs and subsequent clonal evolution.

As illustrated in this study, a fraction of SNVs induced in cancer cells arises in association with SVs; however, in general, induced SBS signatures differ among cancer types^9^. This suggests that different types of SNV may be associated with induction of SVs, and that this will depend on the associated risk factors. To investigate this, we examined the effects of γ-ray irradiation, which accelerates clonal evolution, on SV-associated SNVs, and analyzed the slopes of the linear regressions^17^. We observed that the type of SNV was altered by exposure to irradiation. There was an ~ twofold increase in SNV levels at A:T pairs, but not at G:C pairs; however, such SNVs still occurred in association with SVs (Fig. 6C,D). Thus, the type of SNV induced in association with SVs can be altered by exogenous stresses, such as exposure to radiation.

### SV-associated SNV induction on genes

**Figure 7:**
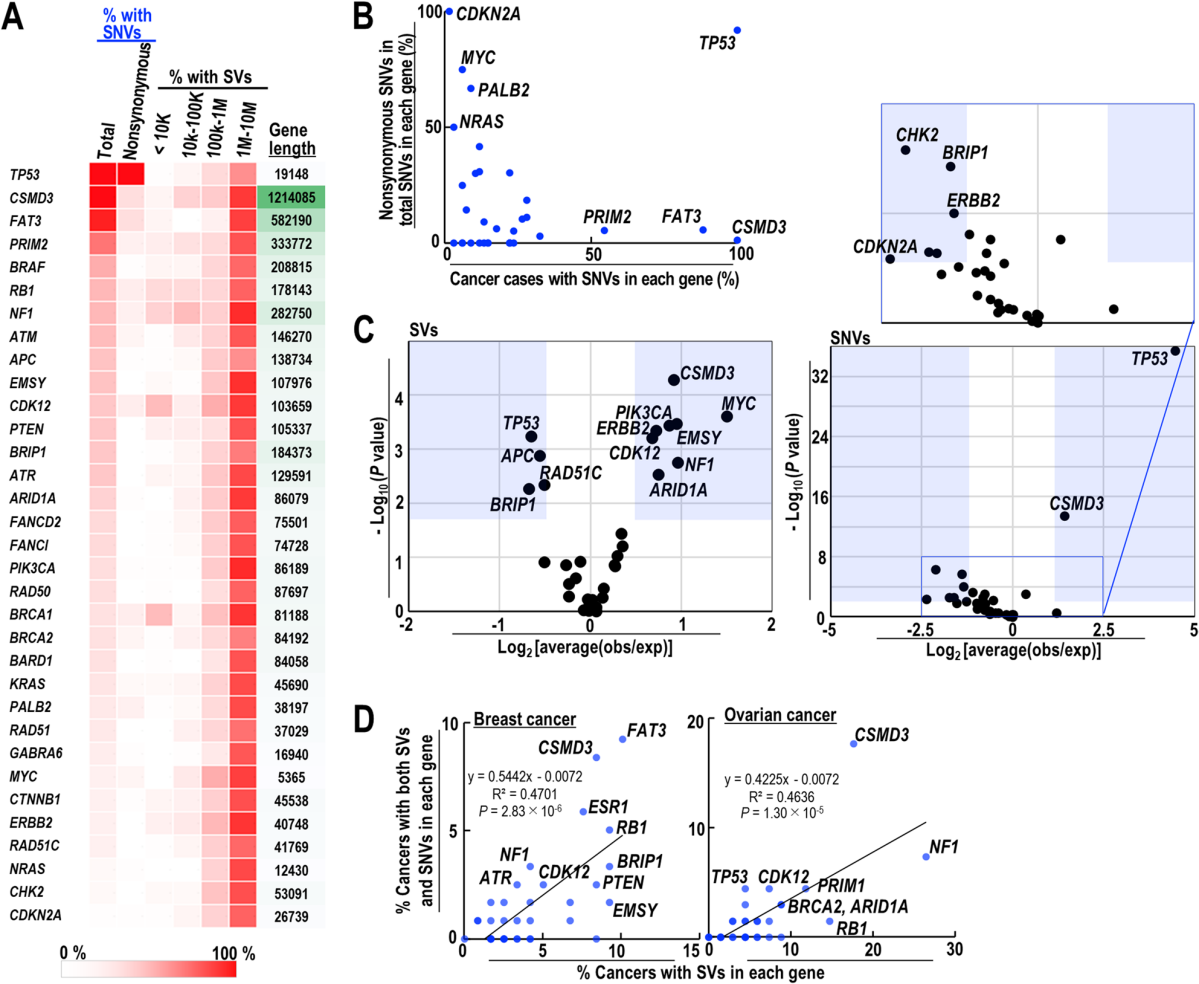
SV-associated induction of SNVs in genes frequently mutated in ovarian cancer. (**A**) Mutation status of genes frequently mutated in ovarian cancer was analyzed. SV status within and outside the genes is shown alongside gene size, in which regions outside genes are analyzed within 10 kb, 10–100 kb, 100 kb–1 Mb, and 1–10 Mb. (**B**) Cancer cases were analyzed by plotting the SNVs in each gene against nonsynonymous SNVs (as a percentage of total SNVs) in each gene. (**C**) Relative SV and SNV statuses of each gene is depicted in volcano plots. (**D**) The number of individual cancer cases harboring both SVs and SNVs was plotted against the number of SVs in each gene (for both breast and ovarian cancers). A two-tailed *t*-test was used for statistical analysis.

To study induction of SVs and SNVs in cancer cell genes, we analyzed genes that are frequently mutated in ovarian cancers (Fig. 7A). First, we observed selective nonsynonymous induction of SNVs in the *TP53* gene of all analyzed cases (Fig. 7B), and found that the data supported such SNVs as cancer-driver mutations. Next, we analyzed enrichment of SVs and SNVs in those genes, and observed two clearly different types (Fig. 7C). One is particularly clear in the *TP53* gene. For this gene, although SVs were induced rarely, nonsynonymous SNVs were present in all tested cases. This implies that although the *TP53* gene is located at a stable genomic locus, SNVs are inevitably induced as a result of selective pressure to drive cancer development. The second type is seen in the *CSMD3* gene, in which induction of both SV and SNV is high; this suggests that this gene is located in an unstable genomic locus. To test whether induction of SV-associated SNVs is a generalized phenomenon in genes, we plotted the number of cancer cases harboring both SVs and SNVs against those harboring SVs in the genes listed in Fig. 7A. As expected, the proportion of genes with both SVs and SNVs increased with the number of SVs, supporting the idea of SV-associated SNV induction in those genes (Fig. 7D). A similar result was observed for breast cancer cells. Taken together, these results suggest that SV-associated induction may be a pathway that generates SNVs in the genes of cancer cells.

## Discussion

The findings of the present study indicate that induction of SNVs in most human cancers is highly associated with SVs. This association was observed in the presence or absence of Kataegis. Kataegis has been defined as localized hypermutation, in which multiple mutations occur within 1 kb windows^11,12^, whereas SV-associated SNV induction, i.e., echoed SV/SNV induction is generally observed across much wider chromosome regions, typically 0.1–10 Mb in size.

Because cancer development is usually induced by SVs and SNVs^23,24^, it is critical to clarify the induction pathways involved. Conventionally, most SNVs are thought to be induced randomly through DNA replication errors, and subsequently accumulate during cell proliferation^4,5^. This appears to occur in pathologically normal organs, as mutation levels generally increase with age^6–8^. However, mutation levels are usually much higher in cancer cells than in healthy tissues^8^ and are often not associated with age (Supplementary Fig. S1C). Rather, the present results indicate that mutagenesis associated with SVs is a major pathway for SNV induction (Fig. 2A). SNVs are highly enriched within 1 Mb of SV regions, and are associated particularly with a type of signature that includes HR deficiency-associated SBS3, exogenous damage-associated SBS7 and SBS36, and APOBEC-associated SBS2 and SBS13 (Fig. 3). These results may suggest a role for genomic instability because HR deficiency and exogenous damage are the direct risk factors. Although clock-like signatures SBS1 and SBS5 also increased in association with SVs (Fig. 2C), the significance is not clear. Since SBS1 and SBS5 are not enriched within 1 Mb of SV regions, the effects are likely to be different from those of SBS signatures associated with HR deficiency, exogenous damage, and APOBEC.

Although it is still unclear how SNVs in cancer cells are induced in a manner associated with SVs, there are at least two possibilities. First, SNVs may increase alongside SVs if the overall level of DNA damage that yields both is high. One example is erroneous repair of DSBs when DNA synthesis is mediated by low-fidelity TLS polymerases, as previously pointed out^16^. Second, SNVs may colocalize with SVs if the regions near the SVs are predisposed to accumulation of SNVs. This might be the case, for example, in cancers with high levels of deaminase (deaminases likely increase the number of localized hypermutation events upon SV induction by exposing single strands of DNA)^25^. These two phenomena can have distinct impacts on induction of SVs and SNVs.

Based on the results of the current study, we suggest that both SNVs and SVs are induced by genomic destabilization-associated mutagenesis; this is because SV-associated SNVs in cancer cells are similar to those in MEFs undergoing clonal evolution caused by genomic instability^16,17^. This argument is supported by the signatures enriched within 1 Mb of the SV regions, as these are associated with defective DNA damage and repair (Fig. 3). Thus, genomic instability-associated SV/SNV induction is a possible pathway that causes SVs and SNVs, although there are probably several other pathways as well. In fact, there are some SNVs that cannot be explained by simple genomic instability-associated induction, e.g., TP53 variations (the majority of which are deleterious SNVs enriched in the DNA binding domain of TP53 in the absence of SVs). Although selective pressure is thought to play a role in TP53 variations (Fig. 7), it is still unclear how these are induced. In addition, although BRCA1 and 2 often harbor germline mutations, it is unclear how such background variations in repair systems affect induction of SVs and SNVs.

The type of mutational signature is affected significantly by the environmental background, e.g., Kataegis is induced by a highly inflammatory background^26^. This is illustrated clearly by our comparison of γ-ray irradiated and non-irradiated cells: although the type of SNVs induced was altered significantly by irradiation, SNVs still correlated strongly with SVs. Importantly, such SV-associated induction of SNVs is associated with induction of SNVs in cancer cell genes (Fig. 7), as well as with the characteristics of the resulting cancer (and its prognosis) (Fig. 5).

## Supplementary Information

Supplementary Information 1.

Supplementary Information 2.

## Data Availability

All data generated or analysed during this study are included in this published article and its supplementary information files.
