# Supplementary material for: Echoed induction of nucleotide variants and chromosomal structural variants in cancer cells
Source: Sci Rep. 2022 Dec 5;12:20964. doi: 10.1038/s41598-022-25479-6 (PMC9723101; doi:10.1038/s41598-022-25479-6)
Supplement: Supplementary file 1 — Supplementary Information 1. [file 41598_2022_25479_MOESM1_ESM.docx]

Supplementary information for

**Echoed Induction of Nucleotide Variants and Chromosomal Structural Variants in Cancer Cells**

Matsuno et al.

**This PDF file includes:**

Supplementary Figures 1–6.


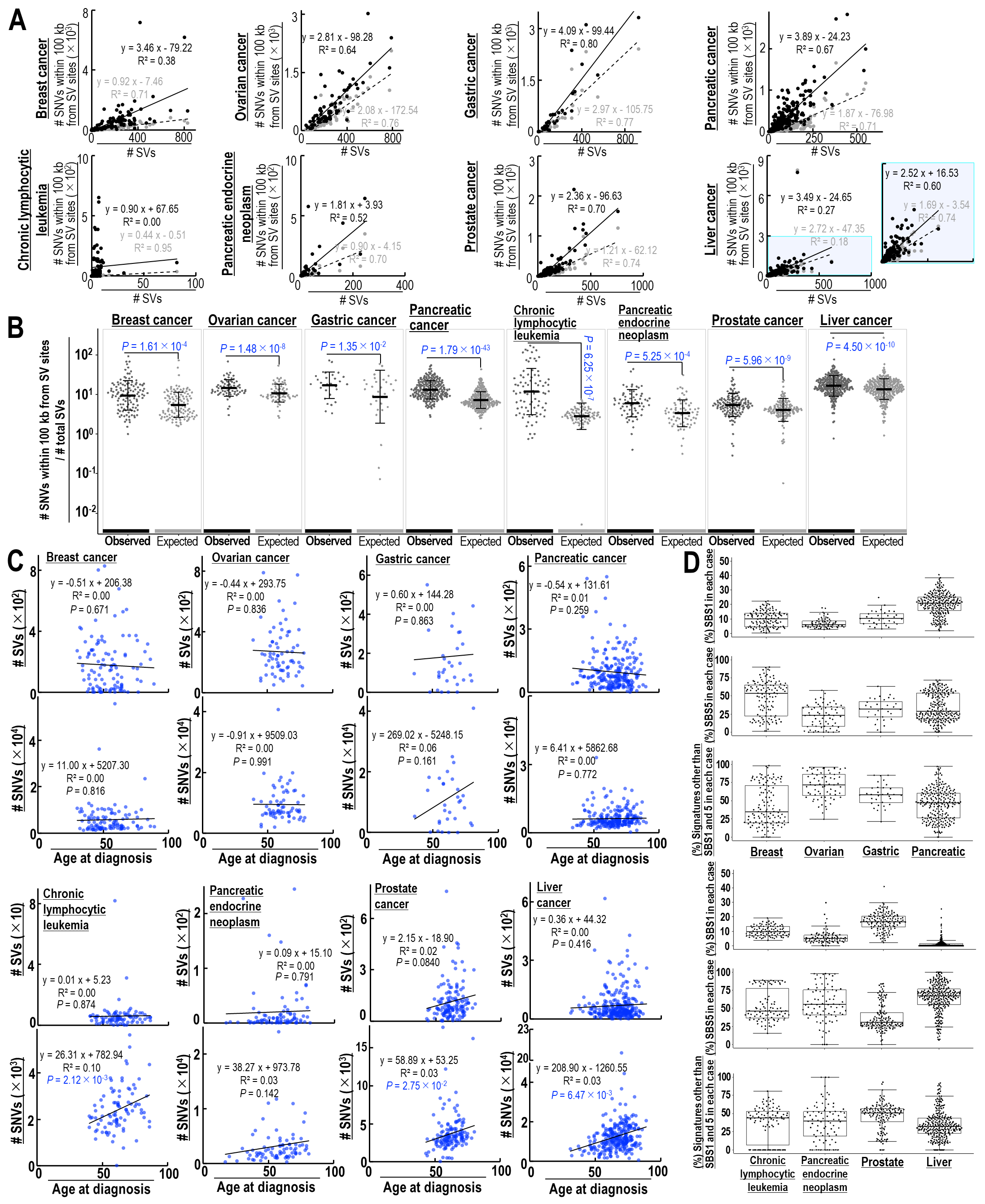


**Supplementary Fig. 1. SV-associated induction of SNV.** **(A, B)** Number of SNVs within 100 kb of each SV site in individual tumors of each type, compared with the random number of SNVs expected **(A)**. The number of expected SNVs was estimated based on a random distribution across detectable SNV sites (blue line) and the whole genome (gray dotted line). SNV counts within 100 kb of SV sites were calculated as a function of the number of SVs induced in individual cancers in each organ. **(B)** Statistical analyses was conducted using two-tailed *t*-tests. **(C)** Number of SVs and SNVs in individual cancers as a function of age at the time of diagnosis. Statistical analyses was conducted using two-tailed *t*-tests. **(D)** Percentage of clock-like signatures (SBS1 and SBS5), and SNVs other than SBS1 and 5, in individual tumors of each type.


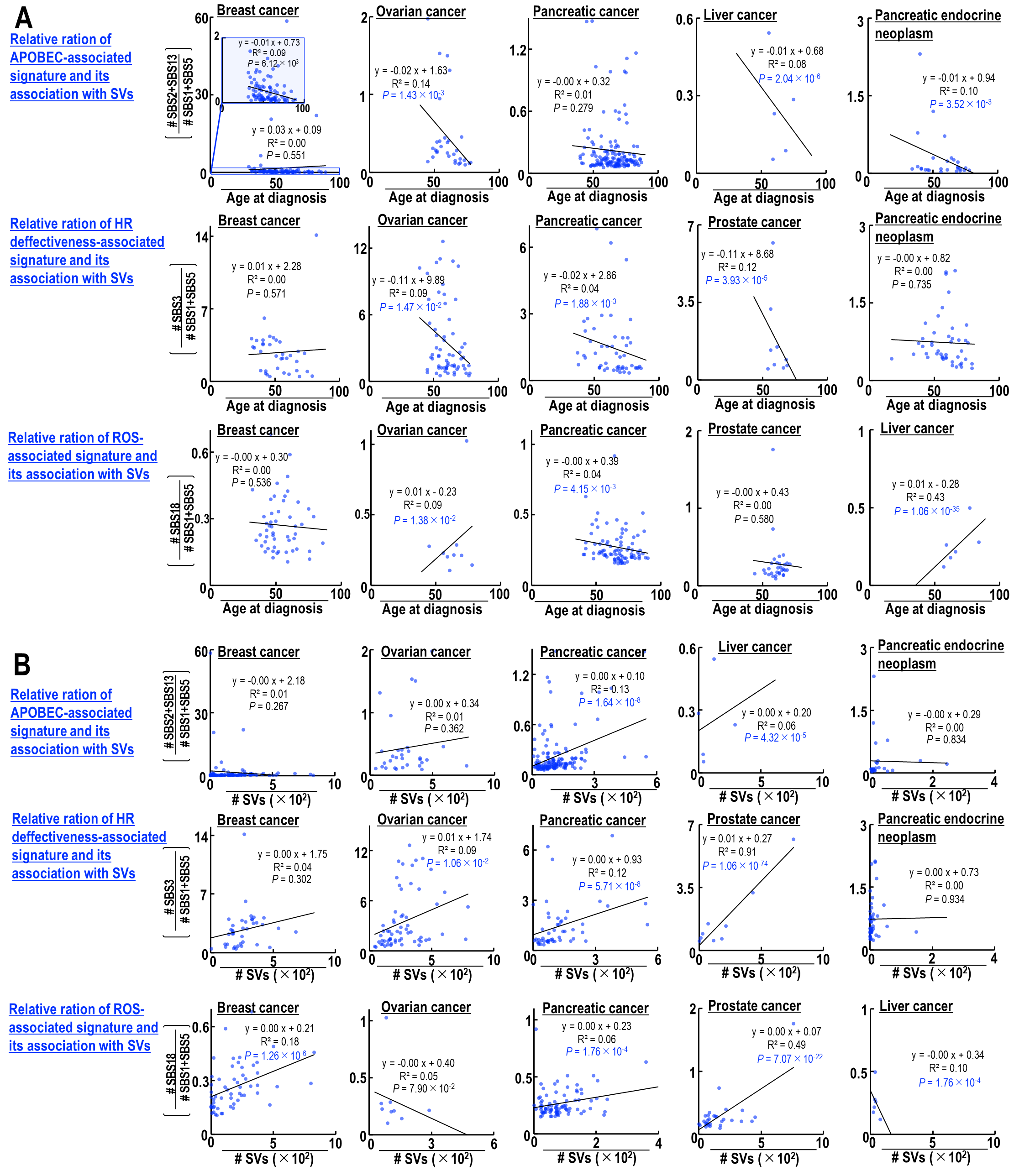


**Supplementary Fig. 2. Mutational signatures not associated with patient age. (A)** Relative number of APOBEC-associated (SBS2+SBS13), HR deficiency-associated (SBS3), and ROS-associated (SBS18) signatures, categorized according to the number of clock-like signatures (SBS1+SBS5), plotted as a function of age at the time of diagnosis of individual patients with each type of cancer. Statistical analyses was conducted using two-tailed *t*-tests. **(B)** Signatures in **(A)** plotted against age at the time of diagnosis of each individual with the indicated cancers, as determined by the relative number of signatures normalized to the number of clock-like signatures (SBS1+SBS5).

**
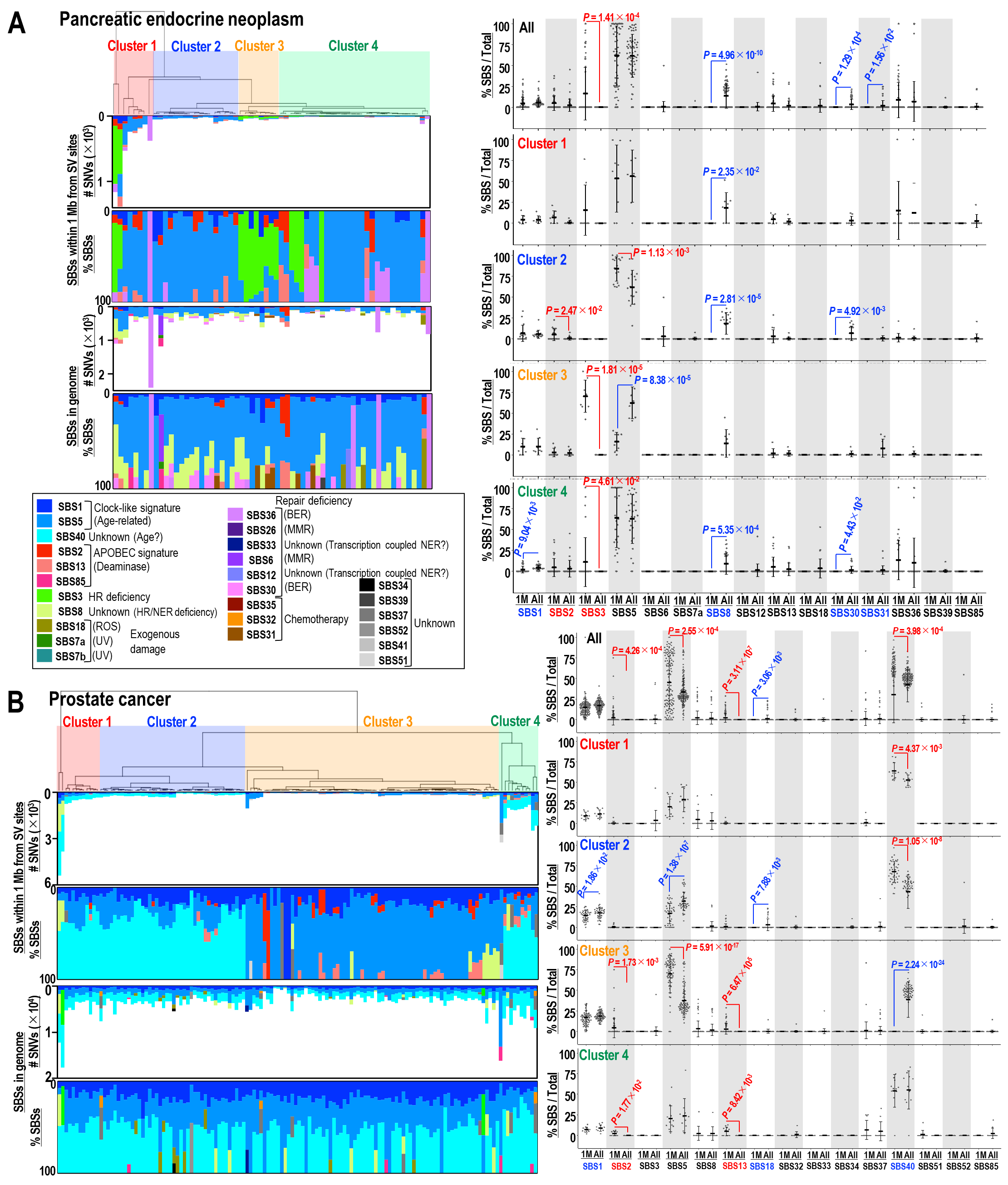
**

**Supplementary Fig. 3. Biased induction of APOBEC- and repair deficiency-associated signatures near SV sites in pancreatic endocrine neoplasms and prostate cancers. (A, B)** Signatures induced in pancreatic endocrine neoplasms **(A)** and prostate cancers **(B)** within 1 Mb of SV sites (left upper panels) and in whole genomes (left bottom panels). The number of signatures was subsequently classified into four clusters. The percentage of each SBS type induced in each tumor was plotted for all tumors and for each cluster (right panels). Statistical analyses by conducted using two-tailed Welch’s *t*-tests.


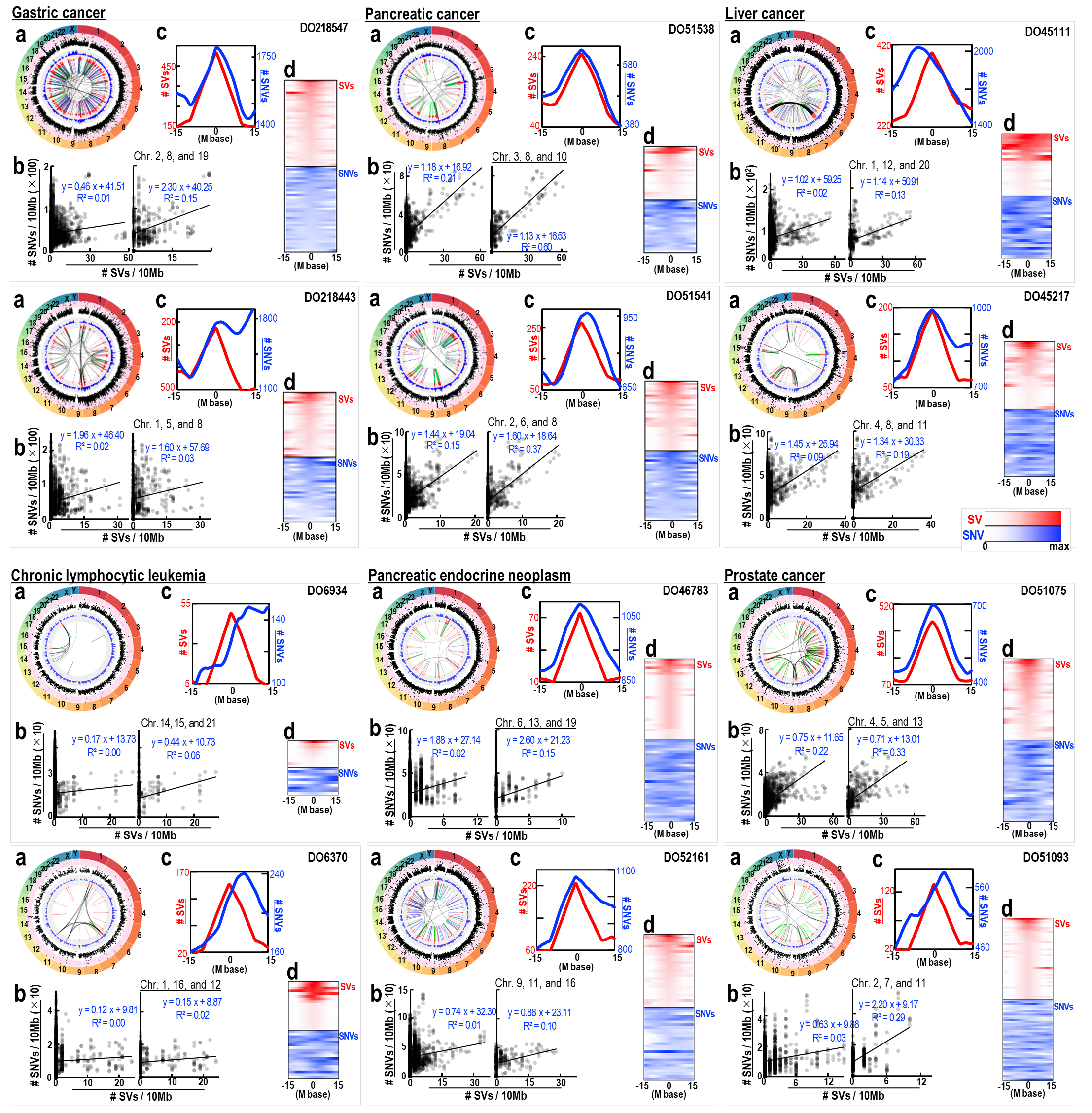


**Supplementary Fig. 4. Inter-SNV distances within SV high and low regions.** SVs and SNVs induced in two individual gastric, pancreatic, liver, and prostate cancers; in chronic lymphocytic leukemia; and in pancreatic endocrine neoplasms. SVs and SNVs in each tumor case were first analyzed by Circos plots **(a)**, with chromosome ideograms shown around the outer ring. The two inner circular tracks show the number of SVs (red) and SNVs (blue), with the corresponding moving averages. The inner lines indicate duplications (blue lines), inversions (green lines), deletions (red lines), and translocations (black lines). SNVs are also shown in rainfall plots, with black dots within pink and white circular zones indicating inter-SNV distances of <1 kb and 1 kb–1 Mb, respectively. Correlations between SV and SNV number in each tumor were plotted in 10 Mb windows **(b)**. Sites at which both the SV and SNV signals in the genome were more than 2-fold higher than the mean were analyzed further by superimposing SV peaks on associated SNV status **(c),** as well as by aligning SV peaks with SNV status **(d)**.


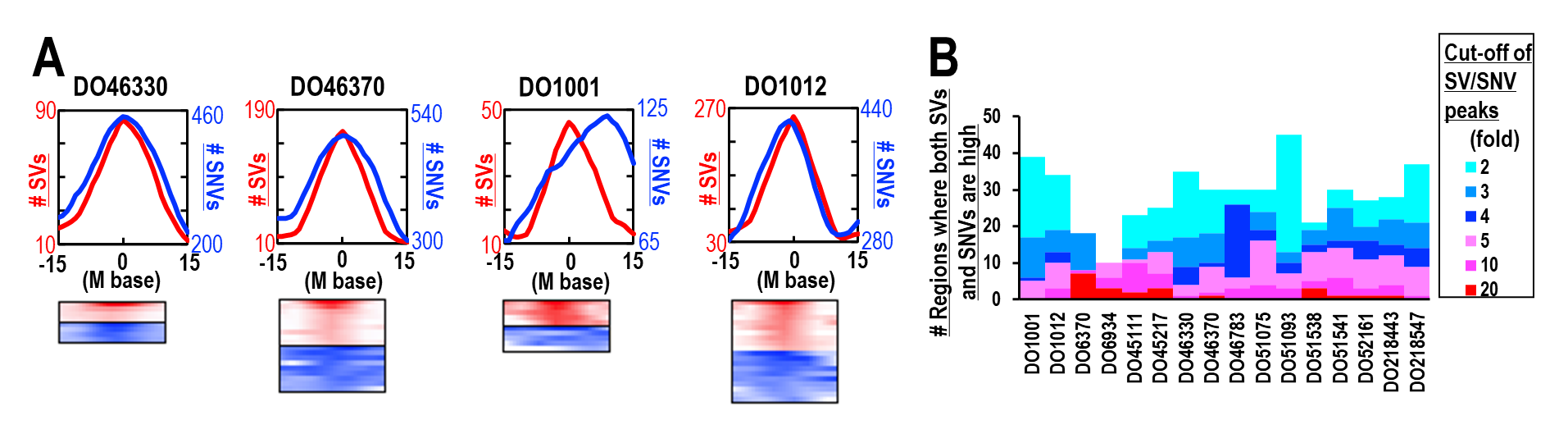


**Supplementary Fig. 5. Echoed induction of SVs and SNVs in individual cancer cases.** **(A)** SVs and SNVs induced in the four cancer cases analyzed in Figure 4A and B were also analyzed to identify those showing more than 5-fold higher induction than the mean in the genome. SV and SNV signals were assessed by summation and superimposition of the SV and SNV data (Top panels), as well as by aligning SV peaks and SNV status (bottom panels). **(B)** Number of regions in which the number of SVs and SNVs was high; there was a different cut-off threshold for each cancer case analyzed in Figure 4 and Supplementary Figure 4.

**
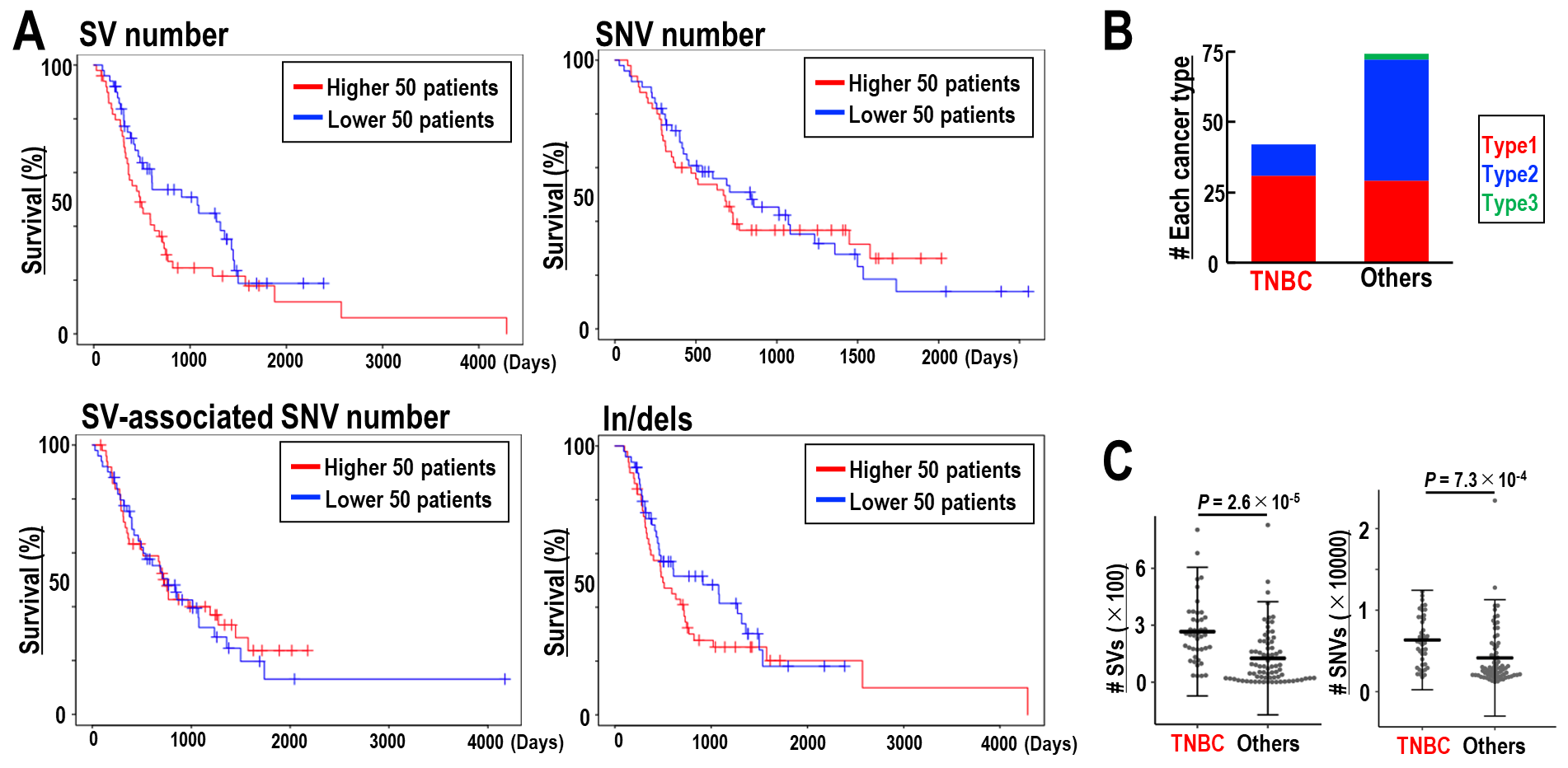
**

**Supplementary Fig. 6. Association between SVs/SNVs and prognosis in patients with pancreatic cancer.** **(A)** Kaplan–Meier analysis of survival of patients with pancreatic cancer stratified by the number of SVs, SNVs, SV-associated SNVs, and indels at the time of diagnosis. Statistical analyses was conducted using log-rank tests. **(B, C)** SV and SNV status of triple-negative breast cancers (TNBCs). The number of SVs and SNVs was analyzed in patients with TNBCs and other types of breast cancer **(B)**. Comparison of the number of SVs and SNVs in types 1 and 2 breast cancer, and in TNBCs and other breast cancers. Statistical analyses was conducted using two-tailed Welch’s *t*-tests **(C)**.
